# Cholangiocarcinoma as an Indication for Liver Transplantation in the Era of Transplant Oncology

**DOI:** 10.3390/jcm9051353

**Published:** 2020-05-05

**Authors:** Enrico Gringeri, Martina Gambato, Gonzalo Sapisochin, Tommy Ivanics, Erica Nicola Lynch, Claudia Mescoli, Patrizia Burra, Umberto Cillo, Francesco Paolo Russo

**Affiliations:** 1 Hepatobiliary Surgery and Liver Transplantation, Padua University Hospital, 35100 Padua, Italy; enrico.gringeri@unipd.it (E.G.); cillo@unipd.it (U.C.); 2 Multivisceral Transplant Unit and Gastroenterology, Padua University Hospital, 35100 Padua, Italy; ericanicolalynch@gmail.com (E.N.L.); burra@unipd.it (P.B.); francescopaolo.russo@unipd.it (F.P.R.); 3 Multi-Organ Transplant and HPB Surgical Oncology, Division of General Surgery, University Health Network, Department of Surgery, University of Toronto, Toronto, ON M5G 2N2, Canada; gonzalo.sapisochin@uhn.ca (G.S.); tommy.ivanics@uhn.ca (T.I.); 4 Surgical Pathology & Cytopathology Unit, Department of Medicine, Padua University Hospital, 35100 Padua, Italy; claudia.mescoli@aopd.veneto.it

**Keywords:** liver transplantation, cholangiocarcinoma, surgery

## Abstract

Cholangiocarcinoma (CCA) arises from the biliary tract epithelium and accounts for 10–15% of all hepatobiliary malignancies. Depending on anatomic location, CCA is classified as intrahepatic (iCCA), perihilar (pCCA) and distal (dCCA). The best treatment option for pCCA is liver resection and when a radical oncological surgery is obtained, 5-year survival rate are around 20–40%. In unresectable patients, following a specific protocol, liver transplantation (LT) for pCCA showed excellent long-term disease-free survival rates. Fewer data are available for iCCA in LT setting. Nevertheless, patients with very early unresectable iCCA appear to achieve excellent outcomes after LT. This review aims to evaluate existing evidence to define the current role of LT in the management of patients with CCA.

## 1. Introduction

Cholangiocarcinoma (CCA) is the second most common hepatic malignancy after hepatocellular carcinoma (HCC); its overall incidence has progressively increased worldwide over the past four decades [1,2,3]. CCA is a malignant tumor of the cholangiocytes (i.e., the epithelial cells lining the bile ducts), which can develop at any portion of the biliary tree, from the canals of Hering to the main bile duct [4,5]. Most CCAs are well, moderately or poorly differentiated adenocarcinomas; other histological subtypes are rarely encountered. Surgery is the preferred therapeutic option for all subtypes of CCA, although, involvement of the vascular structures and lymph nodes needs to be considered when evaluating the best approach.

Refinements in CCA diagnosis and better understanding of genetic profiles may lead to an optimized surgical treatment. In addition, novel drugs, such as checkpoint inhibitors and molecular-targeted molecules, may represent future options for adjuvant and neoadjuvant therapy, alone or in combination with downstaging locoregional therapies [6]. This review aims to analyze the recent advances in the management of CCA, focusing on surgical treatments and highlighting the role of liver transplantation in the era of transplant oncology.

## 2. Anatomo-Pathological Features of CCA

CCAs represent an estimated 3% of all gastrointestinal system malignancies. They are classically divided into three groups, depending on the anatomic site of origin: intrahepatic CCAs (iCCAs), perihilar CCAs (pCCAs) and distal CCAs (dCCAs) [7]. iCCAs are defined as CCAs located proximally to the second-order bile ducts. iCCA can present three different patterns of growth: mass-forming (MF-iCCA), periductal infiltrating (PI-iCCA) and intraductal growing (IG-iCCA). Of these, the MF-iCCA is by far the most frequent type [8]. pCCAs can arise anywhere from the second-order bile ducts to the common bile duct above and at the site of cystic duct insertion. In contrast, dCCAs are confined to the area between the origin of the cystic duct and the ampulla of Vater [7]. pCCAs account for ~50–60% of all CCAs and dCCAs for 20–30% [9]. pCCA and dCCA have similar macroscopic features; they generally are flat or poorly defined nodular sclerosing tumors which diffusely infiltrate into adjacent structures in 80% of cases. Less frequently, pCCAs and dCCAs present as intraductal papillary tumors [10]. PI-iCCAs and flat/nodular sclerosing p/dCCAs often develop from preinvasive lesions, classified as biliary intraepithelial neoplasm (BilIN); likewise, papillary p/dCCAs and IG-iCCA arise from intraductal papillary neoplasms of the bile duct (IPNBs). No preinvasive lesions of the MF-type of iCCA have been identified [11].

Histologically, most pCCAs and dCCAs are mucin-producing adenocarcinomas or papillary tumors, into conventional and unconventional iCCAs. Conventional iCCAs are well to moderately differentiated adenocarcinomas made up of columnar to cuboidal epithelial cells, which bear a resemblance to biliary epithelial cells [12]. Varying degrees of desmoplasia and inflammation can be present. Mucin can be found in the lumen of tubular structures, in the apical side of tumor cells and in the cell cytoplasm. iCCAs cells can cause compression of the surrounding parenchyma or invade hepatocyte plates and sinusoids [10]. iCCAs can also be classified into two main histological subtypes depending on the anatomic site where they may arise [10]. The small bile duct type of iCCA may develop from small intrahepatic bile ducts, progenitor cells or hepatocytes. It presents as small-sized tubular or acinar adenocarcinoma with invasive mass-forming growth pattern and absent or minimal mucin production. It commonly has a peripheral location in the liver [10,13]. On the other hand, large bile duct iCCAs derive from large intrahepatic bile ducts [14] or from peri-biliary glands [15]. They are formed of mucin-producing columnar tumor cells arranged in a large-duct or papillary architecture. Most large bile duct iCCAs are PI-iCAAs and are centrally located in the liver [14,16].

## 3. pCCA: Surgical Approach and LT Indications

### 3.1. Liver Resection

**Figure 1:**
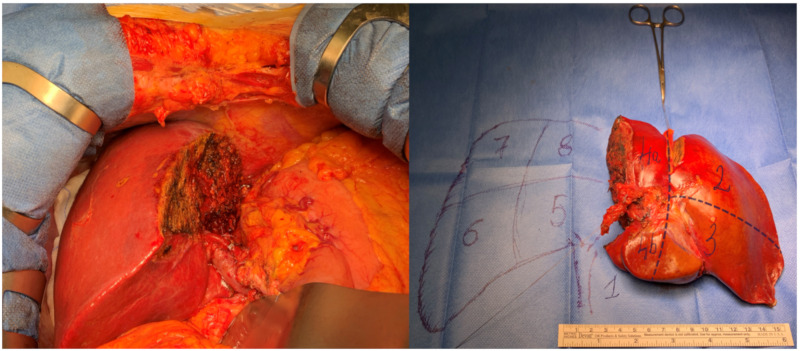
Left hepatectomy with caudate lobe resection for perihilar cholangiocarcinoma (pCCA) (Bismuth IIIb).

When feasible, liver resection (LR) is the best treatment option for pCCA. Surgery can offer a 5-year survival rate of 20–40% after R0 resection (microscopic margins negative for tumor). When R0 is not feasible, the 5-year survival rate drops to 0% [17,18,19,20,21]. Unfortunately, only 20–25% of patients are appropriate candidates for curative resection. Due to its anatomic location—adjacent to the hepatic artery and portal vein—vascular involvement is common in pCCA and represents the main reason for the tumor to be deemed unresectable. Other criteria for unresectability are local progression, jaundice or presence of metastases. The main goal of surgery is to obtain an R0 resection; to achieve it, a complex surgical procedure is required, which involves performing a major hepatectomy with caudate lobe and en-bloc biliary resection, a hepaticojejunostomy and a lymphadenectomy (Figure 1).

Vascular resection and subsequent reconstruction can also be performed, when necessary. Mortality rates remain high even in experienced centers due to the complexity of the procedure. The 30-day mortality rate ranges from 5–15% [22,23,24,25,26,27], and the leading cause of death is postoperative liver failure. An accurate diagnosis is crucial to select patients for surgery correctly. Five to 20% of presumed pCCAs, which are resected, turn out to be negative on histopathological assessment [28] and finally showed benign biliary stricture due to chronic inflammation in the setting of IgG4-disease or primary sclerosis cholangitis (PSC). This means that some patients undergo a high-risk surgical procedure even if no tumor is to be found. Optimal care of patients with pCCA requires the involvement of liver-specific multidisciplinary teams, which should include interventional radiologists, oncologists, hepatologists, endoscopists and hepatobiliary surgeons, with surgical oncology, vascular and transplant skills [29].

#### 3.1.1. Staging

Although there is no universal definition of resectability, there are classifications that can help clinicians choose the best treatment for pCCA. The Bismuth classification was introduced in 1975 and, despite it being the oldest classification, it is still primarily used to define pCCAs according to the biliary segment affected by the tumor. A type IV tumor is characterized by a bilateral involvement of the second-order intrahepatic bile ducts and is generally considered unresectable. However, the Bismuth classification does not describe vascular involvement. In 2001, Blumgart introduced the clinical T staging system, which describes both vascular and parenchymal involvement but does not account for distant metastases or lymph node involvement. Recently, the American Joint Committee on Cancer (AJCC) published the 8th edition of the Cancer Staging Manual [30]. The staging system for pCAA included in the Manual is the most comprehensive, as it provides for the assessment of tumor progression, vascular involvement, lymph node and distant metastases.

#### 3.1.2. Biliary Drainage and Future Liver Remnant Quantification Before LR

Hyperbilirubinemia and low quality/quantity of the future liver remnant (FLR) are two high risk factors for postoperative morbidity and mortality in liver surgery. Both preoperative hyperbilirubinemia and FLR characteristics can, in some cases, be modified before surgery. Obstructive cholangitis is the main indication for biliary drainage before tumor resection. Given the high morbidity of the procedure, the decision whether or not to drain the bile ducts before operating is still debated. Bile duct obstruction causes a pro-inflammatory state, which increases the risk of postoperative morbidity [31]. A recent retrospective study confirmed that a high preoperative bilirubin level is a significant risk factor for postoperative morbidity and mortality. The authors described a cut-off value of 2.5 mg/dL and 6.2 mg/dL for morbidity and mortality, respectively [32]. In the case of major hepatic resection, biliary drainage of the FLR should be strongly recommended [33,34]. There are two biliary drainage procedures: percutaneous transhepatic biliary drainage (PTBD) and endoscopic biliary drainage (EBD). These procedures are associated with a high risk of complications and should be performed by expert hands. Patient with suspected pCCA should be referred to high-volume centers with a multidisciplinary hepatobiliary team [35]. Few studies in the literature compared EBD and PTBD, with conflicting results. Two recent meta-analyses, which were based on retrospective studies, reported a higher conversion rate in the EBD group but similar morbidity in the two groups. Another retrospective study showed higher morbidity after PTBD [36]. The only multicenter randomized clinical trial (RCT) comparing PTBD with EBD before major LR for pCCA was stopped early due to a high mortality rate in the PTBD group (41% vs. 11%) [37]. In a South Korean study, the authors proposed that the use of endoscopic nasobiliary drainage (ENBD) could reduce procedure-related morbidity, despite comparable results between EBD and PTBD [38]. In patients with unresectable pCCA, PTBD with stent placement above the sphincter of Oddi was proposed to reduce bacterial cholangitis. The same procedure should be offered before surgery [39]. In conclusion, EBD should always be preferred, especially when there is still no diagnosis and let the multidisciplinary hepatobiliary team decide based also on the local expertise. Before a major resection, the FLR should always be drained, especially when there is a bile duct obstruction associated with cholangitis, bilirubin levels > 4 mL/dL and FLR < 40%; antibiotic prophylaxis is always indicated; jaundiced patients with suspected pCCA should be referred to specialized centers. Insufficient liver volume and abnormal liver function are significant risk factors for morbidity and mortality after surgery. Post-hepatectomy liver failure (PHLF) is the most frequent cause of death. The FLR is calculated by a CT-scan software depending on the type of operation (extended right or left hepatectomy). An FLR of 25%, if the parenchyma is normal, is considered sufficient. On the other hand, if the liver is “abnormal”—e.g., because of previous chemotherapy, steatosis or cirrhosis—an FLR of 30–40% will be needed [40]. Indocyanine green clearance, hepatobiliary scintigraphy and single-photon emission CT (SPECT)-CT can be used in combination to judge the capacity of the FLR to maintain a normal liver function [41]. A recent multicenter retrospective study showed a significantly lower incidence of liver failure, biliary leakage, abscess formation and 90-day mortality in patients treated with portal vein embolization (PVE) before major LR for pCCA [42].

#### 3.1.3. Surgical Approach

As mentioned before, complete tumor resection with negative margins is the only treatment that can lead to long-term survival. Radiologic preoperative evaluation is crucial for defining resectability and choosing the best surgical approach. Preoperative CT-scans and MRIs can offer substantial information about vascular and biliary involvement. Unfortunately, 45–47% of patients who undergo laparotomy for potentially curative surgery are deemed unresectable intraoperatively due to local progression disease [43,44]. Careful palpation and accurate surgical dissection of the hepato-jejunal ligament are indispensable for defining resectability. The laparoscopic approach does not allow manual palpation but can be useful to exclude local progression and to define resectability, as suggested by Bird et al. [45]. Unsuccessful laparotomy or laparoscopy has a significant impact on prognosis, as previous surgery is an absolute contraindication to liver transplantation.

Surgery for pCCA is very challenging from a technical point of view because of the anatomic location of the tumor. pCCA develops very close to the portal veins and hepatic arteries, therefore performing an R0 resection is not always achievable. Surgery for pCCA consists of a major hepatectomy with caudate lobe and en bloc biliary resection, a hepaticojejunostomy and an accurate lymphadenectomy. Vascular resection and subsequent reconstruction can also be performed when necessary. The choice to perform left or right hemihepatectomy depends on the extension of the tumor and the anatomy of the patient. Choosing right hemihepatectomy has the advantage of the greater length of the left hepatic duct (2–3 cm) when compared with the right hepatic duct (< 1 cm) [46]. The complexity of left hemihepatectomy is due to the difficulty in performing biliary anastomosis to the multiple right hepatic ducts, and especially to the posterior right hepatic duct, as it is located posteriorly to the right portal vein. En-bloc resection of the caudate lobe is always recommended as the tumor typically grows into the caudate lobe through small biliary branches. Thorough anatomic studies have shown that the caudate bile ducts drain into the right or left hepatic ducts or the biliary confluence [47]. A recent retrospective study compared left and right hemihepatectomy for pCCA; left hemihepatectomy had comparable oncologic outcomes to right hemihepatectomy with a lower occurrence of ascites and higher bile leakage rates. However, postoperative ascites was a more notable complication than postoperative bile leakage, as ascites is correlated with postoperative liver function, and all cases of bile leakage that were described were minor complications and resolved spontaneously by conservative care [48]. Outcomes are more favorable in high-volume centers [49].

### 3.2. Liver Transplantation

For unresectable tumors, LT can be considered an option, as it would allow to solve several surgery-related issues (positive margins, inadequate FLR) and also to treat any underlying primary sclerosing cholangitis (PSC). In the past, LT was considered ineffective due to the high rate of recurrence. In the US, the Cincinnati Transplant Tumor Registry published results from 207 patients from 1968 to 1997 who were treated with LT for CCA. The data showed a 5-year survival rate of 23% with a tumor recurrence rate of 51% (84% within the first two years). The survival rate after recurrence was less than one year, and no advantage in survival was detected in patients with CCA and PSC, in patients with incidental tumors or patients receiving adjuvant chemotherapy [50].

In some European countries (Spain, France, Germany), a similar analysis was performed in patients affected by pCCA treated with LT. A 3-year survival rate of 30–38% and a very high rate of recurrence of disease were reported [51,52]. Despite this, some transplant centers enlist patients with pCCA for LT within very restrictive protocols, which involve the association with neoadjuvant chemoradiation treatment.

Innovations in the treatment of pCCA appeared in 1993 when the University of Nebraska and later also the Mayo Clinic liver transplant Center, proposed a new neoadjuvant protocol for the treatment of pCCA [53]; patients affected by pCCA alone or in the setting of PSC were carefully selected for the protocol, which consisted in receiving external beam radiation (40–45 Gy), followed by transcatheter radiation (20–30 Gy) with iridium wires, being administered intravenous 5-fluorouracil for chemo-sensitization during radiation therapy and later capecitabine while awaiting LT (Table 1).

The effectiveness of high-dose neoadjuvant chemo and radiotherapy is well established and LT can improve or resolve the adverse effects connected with the neoadjuvant treatment [54]. Staging surgery with lymph node biopsies is always performed before LT, after brachytherapy, by laparotomy or hand-assisted laparoscopy. Only patients with unresectable pCCAs are eligible for the protocol. The Mayo Clinic group reported an intention-to-treat survival rate at 1, 3 and 5 years of 82%, 62% and 56%, respectively. The overall survival after LT at 1, 3 and 5 years was 91%, 81% and 74%, respectively. Furthermore, patients with PSC had a higher 5-year survival rate than patients with de novo pCCA (80% versus 64%). Recurrence occurred in 17% of patients [55]. These promising data were reproduced in a multicenter study published by Murad in 2012 [56]. Data collected from 12 high-volume centers in the US who met the inclusion criteria for LT were analyzed. The authors reported a 5-years intention-to-treat survival of 53% and an 11.5% drop-out rate after 3.5 months, suggesting an appropriate prioritization for LT and a rigorous selection of the candidates. However, this protocol has been criticized and key issues are still debated.

#### 3.2.1. Diagnostic Accuracy

The main criticism against the Mayo Clinic protocol is that if transperitoneal biopsies cannot be taken or don’t confirm the diagnosis, patients can potentially be transplanted though they may not have cancer; this is suspected when cytology and fluorescent in situ hybridization (FISH) test come out negative and no residual tumor is found in the transplant biopsy specimens.

**Figure 2:**
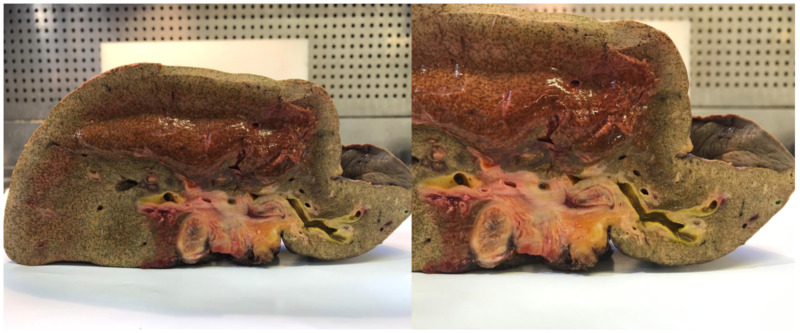
Effect of brachytherapy on bile ducts (explanted liver).

In half of the patients, a pathological diagnosis is not made before neoadjuvant therapy and half of the explanted livers do not have residual CCA at pathological evaluation after LT. Preoperative pathological diagnosis is a challenging task in pCCA. About 15–20% of patients who undergo LR for suspected pCCA are diagnosed with a benign biliary stricture [57]. In this case, the dilemma is: Was the therapy so effective that the tumor disappeared, or was there no tumor in the first place? (Figure 2).

A subgroup analysis of the Mayo Clinic group showed a 66% 5-year overall survival rate when clinicians had reached a pathological diagnosis versus a 92% 5-year overall survival rate in the unconfirmed pathology subgroup. The intention to treat 5-year overall survival rates were 50% and 80% in the confirmed and unconfirmed pathology subgroups, respectively [58].

#### 3.2.2. Prioritization to Liver Transplantation

Prioritization to LT is one of the most debated issues. In 2009, the UNOS Board of Directors agreed on criteria for standardizing model for end-stage liver disease (MELD) score exception points in pCAA and on allowing adjustments every three months. According to data provided by Gores et al. [59], the efficacy of neoadjuvant therapy in highly selected patients affected by unresectable pCCA can justify a MELD score exception for these patients. In the era of transplant survival benefit, the allocation policy may undergo some radical changes.

#### 3.2.3. Effectiveness of Neoadjuvant Therapy or Patient Selection?

Sudan et al., were the first report to describe the favorable outcomes of patients who underwent LT following neoadjuvant radio- and chemotherapy treatment for pCCA [60]. Data published by the Mayo Clinic Group confirmed the efficacy of neoadjuvant radio and chemotherapy before LT for the treatment of pCCA. LT alone for pCCA was considered inadequate due to high recurrence rates and poor patient survival; however, patients had not been strictly selected for LT and most of the cases of LT alone were only described in retrospective studies which covered long timeframes.

Therefore, the question is whether the success of the Mayo Clinic series was due to the careful selection of the patients enrolled in the study or to the efficacy of neoadjuvant therapy. Mantel et al. have focused their attention on careful patient selection. In a retrospective study, they identified 28 patients with pCCA who met the strict selection criteria for the Mayo Clinic protocol but did not undergo neoadjuvant chemoradiation therapy [61]. Five-year survival in this subgroup was 59%, which is comparable to the Mayo Clinic series.

#### 3.2.4. Liver Resection or Liver Transplantation?

Ethun et al., in a retrospective analysis, investigated the efficacy of surgery (LR vs. LT) in patients with pCCA [17]. The overall 5-year survival was 18% and 64% for resected and transplanted patients, respectively. When focusing the analysis on patients meeting the Mayo Clinic transplantation criteria (i.e., tumor smaller than 3 cm, negative lymph nodes) with R0 resection and after excluding PSC, the reported 5-year survival was still higher in the LT group (54% vs. 32%, *p* = 0.049). However, this was only a retrospective study comparing resected patients with transplanted—therefore, unresectable—patients. To date, there are no RCTs comparing LR to LT.

## 4. iCCA: Surgical Approach and LT Indications

### 4.1. Liver Resection

LR remains the best treatment for iCCA [62]. More than 70% of patients with iCCA require a major hepatectomy (defined as resection of ≥ 3 liver segments) to achieve tumor-negative margins [63,64,65,66]. Contraindications to LR include diffuse bi-lobar involvement (satellite lesions), peritoneal carcinomatosis, distant metastases, underlying liver disease (advanced fibrosis, cirrhosis) with portal hypertension, a future liver remnant < 20–30% or inadequate response to portal vein occlusion or severe co-morbidities [67]. Recent studies have demonstrated improved survival rates in patients who receive treatment at academic centers, undergo lymphadenectomy even in node-negative disease and undergo an anatomic rather than a non-anatomic LR [68,69,70]. Lymphadenectomy is recommended by both the National Comprehensive Cancer Network (NCCN) and the International Liver Cancer Association (ILCA), especially for staging and prognosis [63]. The 8th edition of the AJCC Staging Manual [71] recommends that at least six lymph nodes should be collected to achieve a complete nodal staging. However, even though preoperative biopsy is not required before curative surgery, staging laparoscopy helps to identify peritoneal and liver metastases with 36% and 67% of accuracy, respectively and therefore should be considered [72]. A large multicenter study, including 1087 resected iCCA patients with tumor vascular involvement, demonstrated that LR with major vascular resections (i.e., inferior vena cava or portal vein resections) did not portend worse perioperative or oncologic outcomes and could be considered in well-selected patients [73]. Nonetheless, in the setting of locally advanced iCCA, some authors proposed to treat the tumor first and then to evaluate the response to therapy [74]. Neoadjuvant therapy is likely to play a critical role in this setting in the future. Minimally invasive liver surgery has been increasing in the last few years in the US, from 16% in 2010 to ~25% in 2015 [75]. Such minimally invasive-approaches are safe and do not appear to compromise oncological outcomes [76,77]. However, a US study demonstrated lower rates of lymph node sampling compared to open resection [78]. A meta-analysis of 6 studies, including 384 patients who underwent laparoscopic hepatectomy and 2147 patients who underwent open hepatectomy for iCCA showed higher rates of R0 resection in the laparoscopic group with similar perioperative and overall survival [77]. For patients with inadequate predicted postoperative FLR (i.e., < 30% volume in a normal liver or < 50% volume in a cirrhotic liver), surgical techniques such as liver partition and portal vein ligation for staged hepatectomy (ALPPS) and preoperative PVE, can be considered to increase resectability rates [79,80,81]. Nevertheless, few data on the use of these surgical options are available for iCCA. In the largest single-center experience of ALPPS, including 14 patients with iCCA, median overall survival was 64% 4-years after surgery, in patients who completed both phases of the procedure (*n* = 12) [82]. Portal vein embolization showed equivalent FLR hypertrophy in biliary tract cancers compared to hepatocellular carcinoma and colorectal cancers. In a study by Yamashita et al., the authors reported lower complete hepatectomy rates in patients with biliary cancers (*n* = 172, 35% with iCCA) compared with HCC patients (*n* = 70), due to disease progression [83]. Still, acceptable outcomes, both at short- and long-term, were achieved with PVE, regardless of cancer type. Ebata et al. reported data from a large cohort of patients (*n* = 494) with different biliary tract cancers (including CCA and gallbladder cancers) who underwent PVE before extended hepatectomy [84]. They showed that PVE could be considered safe, even in patients with cholestatic liver disease. Three-hundred and seventy-two patients (75%) underwent extended hepatectomy after PVE and achieved long term oncological outcomes (5-year overall survival [OS] 39% in the iCCA group) similar to those reported in iCCA patients after LR [84,85].

### 4.2. Liver Transplantation

Intrahepatic CCA is considered a contraindication for LT in many LT centers due to very poor reported outcomes, with 2-year survival of less than 40% [86,87]. In the last few years, several retrospective studies reported excellent oncologic and survival outcomes after LT in patients with iCCA found at explant pathology [88,89]. The results of LT in iCCA patients can vary, depending on the presence of liver cirrhosis. Sapisochin et al. identified 29 patients who were transplanted for HCC with a radiological diagnosis before LT and iCCA on explant pathology [88]. A subgroup of patients with “very early” iCCA, i.e., tumors ≤ 2 cm, had excellent oncologic outcomes with 1, 3 and 5-year actuarial survival rates of 100%, 73% and 73%, respectively. Poor prognostic factors such as larger tumor size and volume, microvascular invasion and poor differentiation were identified. A multicenter study, including 48 patients, confirmed these findings [90]. A multicenter prospective trial (NCT02878473), currently in the enrollment phase, will further clarify the role of LT in patients with “very early” iCCA. Therefore, given the lack of evidence, LT for iCCA patients with cirrhosis should be performed only in a clinical trial setting. For iCCA patients without cirrhosis, Lunsford et al., in a combined experience with the Houston Methodist and MD Anderson cancer centers, published a prospective case series regarding patients who had locally advanced unresectable iCCA at diagnosis [91]. These patients received neoadjuvant therapy with a first-line platinum-based regimen and gemcitabine and underwent LT after a minimum of 6 months of radiographic response or stability. Among the 21 patients referred, 12 patients agreed to be included in the study, and six patients underwent LT. The 5-year OS and recurrence-free survival rates were 83.3% and 50%, respectively.

Wong et al., presented the results from a pilot study, including a prospective cohort of patients with pCCA and iCCA who underwent downstaging before LT [92]. The study included three iCCA patients and 2 of them underwent LT. The neoadjuvant therapy protocol included locoregional treatment with either stereotactic body radiation or trans-arterial chemoembolization, together with 5-fluorouracil—or capecitabine-based chemotherapy. The inclusion criteria were tumor size ≤ 8 cm and an absence of extrahepatic metastasis. Among the 18 patients included in the study, 11 dropped out (6 patients for tumor progression, and five patients for uncontrolled infection and failure-to-thrive). The recurrence-free survival rate and OS were 80% and 90%, respectively, with a median follow-up of 22.1 months from diagnosis. The definitive role of neoadjuvant therapy before LT, even considering living donor LT as an alternative to resection, remains to be fully elucidated and currently cannot be recommended outside of clinical trials.

## 5. New Frontiers of Systemic Therapies for CCA

In the past decade, CCA genome profiling has been analyzed using high-throughput genomics and specific CCA clusters with different genetic and copy number alterations, gene expression and methylation changes have been identified [93,94]. Two main molecular classes of iCCA were clearly distinguished: the inflammation class, characterized by activated inflammation pathways, with a better outcome and the proliferation class, characterized by the activation of many oncogenic pathways, linked to a worse prognosis [95,96]. All discoveries gave hope for an oncological precision medicine scenario. However, the wide genetic heterogeneity of CCA and its ability to find alternative escapes to date have impaired the response to targeted therapies. There are currently many ongoing trials investigating the efficacy and safety of several promising targeted therapies, especially those combining different molecules.

### 5.1. Cytotoxic Chemotherapy

Surgery is the primary curative treatment option for iCCA at an early stage. Nevertheless, due to the high recurrence rates after resection, effective adjuvant therapy is needed to improve recurrence-free survival (RFS) [6]. Systemic therapy can be combined with surgery, depending on tumor location, T and N stage, as well as resection margins. Studies of adjuvant treatment options have historically been small, retrospective and non-randomized and include patients with all types of biliary tract cancers (BTC). The PRODIGE study [97] included 194 patients with iCCA (46%), pCCA (8%), dCCA (27%) or gallbladder cancer (20%). Similar percentages of patients with involved lymph nodes and R0 resection (> 80%) rates were present in the gemcitabine and oxaliplatin group compared to the surveillance group. Between the two groups, no significant differences were seen for the primary outcomes, including RFS at 12 and 24 months. Similarly, Ebata et al. [84] showed no significant difference in RFS or OS in a study which included patients with extrahepatic CCA (45% hilar, 55% distal). Recently, results from randomized controlled trials of adjuvant therapy for BTC comparing cytotoxic chemotherapy options with observation have been reported. The BILCAP study [98], including 447 patients with iCCA (19%), hilar CCA (29%), gallbladder cancer (18%) or dCCA (35%), found a significant difference in overall survival in the per-protocol analysis in favor of capecitabine versus no drug (53 months for capecitabine group and 36 months for controls). According to ASCO 2019, with moderate evidence quality and moderate strength of recommendation, patients who underwent BTC resection should receive adjuvant capecitabine chemotherapy for a duration of 6 months [99]. As expected, those patients with a smaller number of LN involved and margin resection R0, survived longer. The identification of different subgroups showing different treatment response can be used to design future trials in order to obtain better results. Regarding patients who cannot undergo surgery for advanced BTC, including iCCA, the current first-line standard-of-care chemotherapy is the combination of gemcitabine plus cisplatin (GemCis) or other platinum-based agents [99] with a median survival rate of less than one year. According to the ABC-02 study [100], patients who received GemCis had a 3-month higher survival rate than those who were administered gemcitabine alone. These data were confirmed by other studies [101] and a meta-analysis [102] confirmed these findings. Unfortunately, the addition of other drugs to GemCis (merestinib or ramucirumab) [103] cediranib [104] and cetuximab [105] have not had a significant impact on survival rates. The role of second-line chemotherapy remains unclear [106,107,108] and only less than 15% of patients are eligible for it, due to rapidly worsening clinical status. For most patients, active symptom control (ASC) is considered the standard of care after progression during first-line chemotherapy. However, small prospective and retrospective studies have shown signs of potential benefits in selected patients [107,108]. The ABC-06 study [109] randomized patients to ASC alone or ASC with oxaliplatin/5-FU chemotherapy (ASC+mFOLFOX) for patients with locally advanced/metastatic BTC previously treated with GemCis. ASC+mFOLFOX improved OS with a clinically meaningful increase in 6 months and 12 months OS rate. In a post hoc analysis [110] of prospective, randomized trials ABC-01, -02 and -03 including patients with advanced BTC. These studies explored the role of first-line systemic chemotherapy in advanced BTCs (cisplatin and gemcitabine vs gemcitabine (ABC-01 and ABC-02) and cisplatin-gemcitabine-cediranib vs cisplatin-gemcitabine-placebo (ABC-03)). Patients diagnosed with advanced iCCA had a better OS compared with other BTCs; a similar trend was identified for patients diagnosed with liver-only iCCA. These findings should be considered for future clinical trials design.

### 5.2. Targeted Chemotherapy

The molecular analysis of CCA by next generation sequencing revealed that two-thirds of patients harbored genomic alterations that could be targeted by specific therapies and therefore, could be used to select patients for different treatments [111]. One of the most explored mutations in CCA are fibroblast growth factor receptor (FGFR) fusions, which are present in 11% to 45% of patients according to different series and may have a high therapeutic impact in the future [112]. In a phase II clinical trial, a FGFR, BJG398, was used in 61 previously treated patients with advanced or metastatic intrahepatic or extrahepatic CCA. FGFR rearrangements were present in 48 out of 61 patients. Overall, response rate was 14.8% (complete + partial response) and overall median progression free survival was 5.8 months, disease control rate (DCR) was 83.3% with median disease duration of 7 months [113]. Mazzaferro et al. [114] reported encouraging anti-tumor activity and an acceptable safety profile in patients with advanced, unresectable iCCA with FGFR2 fusion, who progressed after chemotherapy and treated with derazantinib, an orally bioavailable, multi-kinase inhibitor with potent pan-FGFR activity.

Recently, data from a multicenter, open-label, single-arm, multicohort, phase 2 study (FIGHT-202) [115] supported the second-line use of pemigatinib in patients with locally advanced or metastatic CCA and FGFR2 fusions or rearrangements. The authors showed that 35% of patients with FGFR2 fusions or rearrangements achieved an objective response and responses were durable. No patients with other FGF/FGFR alterations or no FGF/FGFR alterations achieved a response. Progression-free survival at 6 months was higher in patients with FGFR2 fusions compared to patients with other or no FGF/FGFR alterations (62% vs 25% and 6%, respectively). Hyperphosphatemia was the most common all-grade adverse event irrespective of cause (88 out of 146 patients (60%)), 45% patients had serious adverse events and 49% of patients died during the study, most frequently because of disease progression. Overall, some FGFR inhibitors are better than others because of resistance clone development, as reported by Goyal et al. [116]. Several competing phase III studies using FGFR vs. GemCis are ongoing in patients with naïve and advanced CCA (NCT03656536, NCT03773302, NCT04093362).

Another specific targetable genetic aberration expressed by CCA is the isocitrate dehydrogenase (IDH) -1 and -2 mutations [94], the IDH-1 mutation being more common than IDH-2 mutation. IDH-1 mutation is druggable and its inhibitor, AG-120 (ivosidenib), reduced the risk of progression or death by 63% compared with placebo for pre-treated patients with IDH1-mutant advanced CCA, according to findings from the global, phase 3, multicenter, double-blind, randomized study of AG-120 (ClarIDHy study) [117]. The median progression-free survival was 2.7 versus 1.4 months for ivosidenib and placebo, respectively. Even if there was not a statistically significant improvement in overall survival, the significant improvement in progression free survival (PFS), the favorable overall survival (OS) trend and the tolerable safety profile all support the clinical benefit of ivosidenib in patients with IDH1-mutated CCA. Other IDH-1 and IDH-2 inhibitors (NCT02273739, NCT02381886, NCT02481154) are currently being tested in ongoing clinical trials.

### 5.3. Immunotherapy

CCA is a cancer with a rich tumor stroma, so the interest in immunotherapy for CCA has increased over the last few years [12]. The first experience with immune checkpoint inhibitors (ICIs) in CCA was the Keynote-028 study [118], a phase 1 multicenter study using pembrolizumab in 24 selected patients with programmed death-ligand 1 (PD-L1) positive advanced CCA or gallbladder cancer after failure to respond to standard therapies. They achieved partial response in 17% of patients enrolled in the study. Bang et al. [119] presented the results from 2 studies, including a cohort of 104 patients with BTC, without a specific differentiation. More than 50% were PDL-1 positive, and nearly all of them had proficient mismatch repair (pMMR). Overall, partial response was achieved in 7% of PD-L1+ patients and median PFS and OS of 2 and 7 months, respectively. Le at al. [120] evaluated the efficacy of PD-1 blockade in patients with advanced mismatch repair-deficient solid tumors (dMMR), including 4 CCA patients. Objective radiographic responses were observed in 53% of patients, and complete responses were achieved in 21% of patients with dMMR. As a result, the US FDA approved pembrolizumab for dMMR/MSI-high solid tumors in 2017. Even when ICIs are combined with different approaches, such as chemotherapies, the efficacy is limited. Ikeda et al. [121] reported results from 30 patients with BTC who received nivolumab and GemCis (iCCA 50%, dCCA 17%, gallbladder cancer 33%). Partial response was 37% and median OS was 15 months. However, more than 90% of patients presented grade ≥ 3 of adverse events related to therapy. Another therapeutic approach was to combine ICIs and a targeted drug. For example, anti-vascular endothelial growth factor receptor 2 (VEGFR-2) monoclonal antibody was used with pembrolizumab in a multicenter phase 1 trial involving 26 patients with different BTCs, showing a very low median PFS of less than 2 months [122]. Similar PFR was obtained using a combination of ICIs, durvalumab (D; anti-PD-L1 mAb) and tremelimumab (T; anti-CTLA-4 mAb), in a small group of patients with BTC. Median response duration for the D cohort was 9.7 months, and 8.5 months for the D+T cohort. Median overall survival was 8.1 months and 10.1 months for the D and D+T cohorts, respectively. One death due to treatment-related adverse events (drug-induced liver injury) was reported in the D+T groups, none in the D cohort [123]. To date, the response rate to ICIs is low across the different trials, but durable and more data are needed to identify those patients who may achieve higher response rates. Among the candidate biomarkers of response to immune-checkpoint inhibition, the most studied biomarker was PD-L1, as in some tumors it was associated with sensitivity to ICI treatment. However, PD-L1 quantitation for immunotherapy response prediction is limited, and there is a need for better response biomarkers [124]. The presence of tumor-infiltrating lymphocytes (TILs) [125] and immune gene expression signatures represent emerging predictive biomarkers. In recent years, considerable effort has been made to identify non-invasive biomarkers with high-throughput omics technologies [126]. An innovative, non-invasive method to identify diagnostic and prognostic biomarkers could be the liquid biopsy. The detection of circulating tumor cells, coding mRNA, cell-free DNA, extracellular vesicles (VE) will be crucial on many fronts, such as early prognosis, prediction of treatment response and chemoresistance. In this perspective, the earlier the diagnosis of CCA, the higher the chance will be for patients to undergo LR or LT, achieving excellent survival rates and undoubtedly improving their prognosis.

## 6. Conclusions

In the era of transplant oncology, LT represents a good option for unresectable pCCA. The introduction of direct-acting antivirals (DAAs) has surprisingly changed the prognosis of patients with HCV infection [127]. This efficacy has resulted in a progressive drop in the number of patients listed for LT, making livers available for other indications. In the context of this new scenario, the dilemma is: is it correct to use this resource to extend LT indications or should we reduce the discrepancy between the number of donors and the number of patients enlisted for LT?

LR represents the best treatment for pCCA when R0 liver resection is feasible. In unresectable tumors, the median survival is 15 months compared with LT that can offer an overall 5-year survival rate of more than 65%; transplantation therefore constitutes the ideal treatment for patients with unresectable pCCA. Patients with pCCA should be referred to high-volume liver transplant centers.

Unresectable very-early iCCA showed good outcomes after LT, but more data from clinical trials are needed, including the combination of LT with systemic neoadjuvant regimens.

When the tumor is in an advanced stage and liver resection and liver transplantation are not feasible, systemic therapy represents the only available treatment for both pCCA and iCCA.

Given that CCA is considered a rare disease, the collaboration of international consortia is needed (e.g., TIGER-LC, ENS-CCA, Cholangiocarcinoma Foundation) to gather large-scale data.

## Figures and Tables

**Table 1:** Mayo Clinic Protocol.

Mayo Clinic Protocol	External beam radiation therapy (45 Gy in 30 fractions, 1.5 Gy twice daily)
	Brachytherapy (20 Gy at 1 cm in approximately 20–25 h)—administered 2 weeks following completion of external beam radiation therapy
	Capecitabine—administered until the time of transplantation, held during perioperative period for staging
	Abdominal exploration for staging—as time nears for deceased donor transplantation or day prior to living donor transplantation
	Liver transplantation
Inclusion Criteria	Diagnosis of pCCA (transcatheter biopsy or brush cytology, CA 19–9 > 100 mg/mL and/or a mass on cross-sectional imaging with a malignant appearing stricture on cholangiography)
	Unresectable tumor above cystic duct (pancreatoduodenectomy for microscopic involvement of CBD, resectable pCCA arising in PSC)
	Radial tumor diameter 3 cm
	Absence of intrahepatic and extrahepatic metastases
	Candidate for liver transplantation
Exclusion Criteria	Intrahepatic cholangiocarcinoma
	Uncontrolled infection
	Prior radiation or chemotherapy
	Prior biliary resection or attempt resection
	Intrahepatic metastases
	Evidence of extrahepatic disease
	History of other malignancy within 5 years
	Transperitoneal biopsy (including percutaneous and EUS-guided FNA)

pCCA: perihilar cholangiocarcinoma; PSC: primary sclerosis cholangitis, CA19-9: Carbohydrate Antigen 19-9; CBD: common bile duct; EUS: endoscopic ultrasound; FNA: guided fine-needle aspiration.

## References

[1] Saha S.K., Zhu A.X., Fuchs C.S., Brooks G. (2016). Forty-Year Trends in Cholangiocarcinoma Incidence in the U.S.: Intrahepatic Disease on the Rise. Oncology.

[2] Khan S.A., Taylor-Robinson S.D., Toledano M.B., Beck A., Elliott P., Thomas H.C. (2002). Changing international trends in mortality rates for liver, biliary and pancreatic tumours. J. Hepatol..

[3] Taylor-Robinson S.D., Toledano M.B., Arora S., Keegan T., Hargreaves S., Beck A., Khan S., Elliott P., Thomas H.C. (2001). Increase in mortality rates from intrahepatic cholangiocarcinoma in England and Wales 1968–1998. Gut.

[4] Burden G. (2015). The Global Burden of Cancer 2013. JAMA Oncol..

[5] Nakeeb A., Pitt H.A., Sohn T.A., Coleman J., Abrams R.A., Piantadosi S., Hruban R.H., Lillemoe K.D., Yeo C.J., Cameron J.L. (1996). Cholangiocarcinoma. A spectrum of intrahepatic, perihilar, and distal tumors. Ann. Surg..

[6] Mazzaferro V., Gorgen A., Roayaie S., Busset M.D.D., Sapisochin G. (2020). Liver resection and transplantation for intrahepatic cholangiocarcinoma. J. Hepatol..

[7] Rizvi S., Khan S.A., Hallemeier C.L., Kelley R.K., Gores G.J. (2017). Cholangiocarcinoma—Evolving concepts and therapeutic strategies. Nat. Rev. Clin. Oncol..

[8] Blechacz B., Komuta M., Roskams T., Gores G.J. (2011). Clinical diagnosis and staging of cholangiocarcinoma. Nat. Rev. Gastroenterol. Hepatol..

[9] DeOliveira M.L., Cunningham S.C., Cameron J.L., Kamangar F., Winter J.M., Lillemoe K.D., Choti M.C., Yeo C.J., Schulick R.D. (2007). Cholangiocarcinoma—Thirty-one-year experience with 564 patients at a single institution. Ann. Surg..

[10] Nakanuma Y., Kakuda Y. (2015). Pathologic classification of cholangiocarcinoma: New concepts. Best Pr. Res. Clin. Gastroenterol..

[11] Kendall T., Verheij J., Gaudio E., Evert M., Guido M., Goeppert B., Carpino G. (2019). Anatomical, histomorphological and molecular classification of cholangiocarcinoma. Liver Int..

[12] Bañales J., Cardinale V., Carpino G., Marzioni M., Andersen J.B., Invernizzi P., Lind G.E., Folseraas T., Forbes S.J., Fouassier L. (2016). Cholangiocarcinoma: Current knowledge and future perspectives consensus statement from the European Network for the Study of Cholangiocarcinoma (ENS-CCA). Nat. Rev. Gastroenterol. Hepatol..

[13] Sia D., Villanueva A., Friedman S.L., Llovet J.M. (2017). Liver Cancer Cell of Origin, Molecular Class, and Effects on Patient Prognosis. Gastroenterology.

[14] Komuta M., Govaere O., Vandecaveye V., Akiba J., Van Steenbergen W., Verslype C., Laleman W., Pirenne J., Aerts R., Yano H. (2012). Histological diversity in cholangiocellular carcinoma reflects the different cholangiocyte phenotypes. Hepatology.

[15] Cardinale V., Wang Y., Carpino G., Reid L.M., Gaudio E., Alvaro D. (2012). Mucin-producing cholangiocarcinoma might derive from biliary tree stem/progenitor cells located in peribiliary glands. Hepatology.

[16] Nakanuma Y., Xu J., Harada K., Sato Y., Sasaki M., Ikeda H., Kim J., Yu E. (2011). Pathological spectrum of intrahepatic cholangiocarcinoma arising in non-biliary chronic advanced liver diseases. Pathol. Int..

[17] Ethun C.G., Lopez-Aguiar A.G., Anderson U.J., Adams A.B., Fields R.C., Doyle M.B., Chapman W.C., Krasnick B.A., Weber S.M., Mezrich J.D. (2018). Transplantation Versus Resection for Hilar Cholangiocarcinoma: An Argument for Shifting Treatment Paradigms for Resectable Disease. Ann. Surg..

[18] Nagino M. (2018). Surgical Treatment of Perihilar Cholangiocarcinoma: Resection or Transplant?. Ann. Surg..

[19] Rosen C.B. (2018). Transplantation Versus Resection for Hilar Cholangiocarcinoma: An Argument for Shifting Paradigms for Resectable Disease in Annals of Surgery 2018. Ann. Surg..

[20] Vibert E., Boleslawski E. (2019). Transplantation Versus Resection for Hilar Cholangiocarcinoma. Ann. Surg..

[21] Resch T., Esser H., Cardini B., Schaefer B., Zoller H., Schneeberger S. (2018). Liver transplantation for hilar cholangiocarcinoma (h-CCA): Is it the right time?. Transl. Gastroenterol. Hepatol..

[22] Endo I., Matsuyama R., Taniguchi K., Sugita M., Takeda K., Tanaka K., Shimada H. (2011). Right hepatectomy with resection of caudate lobe and extrahepatic bile duct for hilar cholangiocarcinoma. J. Hepato Biliary Pancreatic Sci..

[23] Launois B., Reding R., Lebeau G., Buard J.L. (2000). Surgery for hilar cholangiocarcinoma: French experience in a collective survey of 552 extrahepatic bile duct cancers. J. Hepato Biliary Pancreatic Surg..

[24] Lee S.G., Lee Y.J., Park K.-M., Hwang S., Min P.C. (2000). One hundred and eleven liver resections for hilar bile duct cancer. J. Hepato Biliary Pancreatic Surg..

[25] Jarnagin W.R., Fong Y., Burke E.C., Bodniewicz J., Youssef M., Klimstra D., Blumgart L.H., DeMatteo R.P., Gönen M. (2001). Staging, Resectability, and Outcome in 225 Patients With Hilar Cholangiocarcinoma. Ann. Surg..

[26] Tsao J.I., Nimura Y., Kamiya J., Hayakawa N., Kondo S., Nagino M., Miyachi M., Kanai M., Uesaka K., Oda K. (2000). Management of hilar cholangiocarcinoma—Comparison of an American and a Japanese experience. Ann. Surg..

[27] Saldinger P.F., Blumgart L.H. (2000). Resection of hilar cholangiocarcinoma-a European and United States experience. J. Hepato Biliary Pancreatic Surg..

[28] Van Gulik T., Kloek J., Ruys A.T., Busch O., Van Tienhoven G., Laméris J.S., Rauws E.A.J., Gouma D.J. (2011). Multidisciplinary management of hilar cholangiocarcinoma (Klatskin tumor): Extended resection is associated with improved survival. Eur. J. Surg. Oncol. (EJSO).

[29] Seyama Y., Kubota K., Sano K., Noie T., Takayama T., Kosuge T., Makuuchi M. (2003). Long-Term Outcome of Extended Hemihepatectomy for Hilar Bile Duct Cancer With No Mortality and High Survival Rate. Ann. Surg..

[30] American Joint Committee on Cancer (2017). AJCC: Cancer Staging Manual.

[31] Kimmings A.N., Van Deventer S.J., Obertop H., Rauws E.A., Gouma D.J. (1995). Inflammatory and immunologic effects of obstructive jaundice: Pathogenesis and treatment. J. Am. Coll. Surg..

[32] Wronka K.M., Grąt M., Stypułkowski J., Bik E., Patkowski W., Krawczyk M., Zieniewicz K. (2019). Relevance of Preoperative Hyperbilirubinemia in Patients Undergoing Hepatobiliary Resection for Hilar Cholangiocarcinoma. J. Clin. Med..

[33] Ba Y., Yue P., Leung J.W., Wang H., Lin Y., Bai B., Zhu X., Zhang L., Zhu K., Wang W. (2020). Percutaneous transhepatic biliary drainage may be the preferred preoperative drainage method in hilar cholangiocarcinoma. Endosc. Int. Open.

[34] Neuhaus H. (2020). Preoperative biliary drainage in hilar cholangiocarcinoma: When and how?. Endosc. Int. Open.

[35] Dumonceau J.-M., Tringali A., Papanikolaou I.S., Blero D., Mangiavillano B., Schmidt A., Vanbiervliet G., Costamagna G., Devière J., García-Cano J. (2018). Endoscopic biliary stenting: Indications, choice of stents, and results: European Society of Gastrointestinal Endoscopy (ESGE) Clinical Guideline - Updated October 2017. Endoscopy.

[36] Kishi Y., Shimada K., Nara S., Esaki M., Kosuge T. (2016). The type of preoperative biliary drainage predicts short-term outcome after major hepatectomy. Langenbecks Arch. Surg..

[37] Coelen R., Roos E., Wiggers J.K., Besselink M.G., Buis C.I., Busch O.R., DeJong C.H., Van Delden O.M., Van Eijck C.H., Fockens P. (2018). Endoscopic versus percutaneous biliary drainage in patients with resectable perihilar cholangiocarcinoma: A multicentre, randomised controlled trial. Lancet Gastroenterol. Hepatol..

[38] Jo J.H., Chung M.J., Han D.H., Park S.W., Bang S., Song S.Y. (2016). Best options for preoperative biliary drainage in patients with Klatskin tumors. Surg. Endosc..

[39] Kubota K., Hasegawa S., Iwasaki A., Sato T., Fujita Y., Hosono K., Nakajima A., Mori R., Matsuyama R., Endo I. (2016). Stent placement above the sphincter of Oddi permits implementation of neoadjuvant chemotherapy in patients with initially unresectable Klatskin tumor. Endosc. Int. Open.

[40] Shoup M. (2003). Volumetric Analysis Predicts Hepatic Dysfunction in Patients Undergoing Major Liver Resection. J. Gastrointest. Surg..

[41] De Graaf W., Van Lienden K.P., Van Gulik T., Bennink R.J. (2010). 99mTc-Mebrofenin Hepatobiliary Scintigraphy with SPECT for the Assessment of Hepatic Function and Liver Functional Volume Before Partial Hepatectomy. J. Nucl. Med..

[42] Olthof P.B., Aldrighetti L., Alikhanov R., Cescon M., Groot Koerkamp B., Jarnagin W.R., Nadalin S., Pratschke J., Schmelze M., Sparrelid E. (2020). Portal Vein Embolization is Associated with Reduced Liver Failure and Mortality in High-Risk Resections for Perihilar Cholangiocarcinoma. Ann. Surg. Oncol..

[43] Ito F., Agni R., Rettammel R.J., Been M.J., Cho C.S., Mahvi D.M., Rikkers L.F., Weber S.M. (2008). Resection of hilar cholangiocarcinoma—Concomitant liver resection decreases hepatic recurrence. Ann. Surg..

[44] Matsuo K., Rocha F.G., Ito K., D’Angelica M.I., Allen P.J., Fong Y., DeMatteo R.P., Gönen M., Endo I., Jarnagin W.R. (2012). The Blumgart Preoperative Staging System for Hilar Cholangiocarcinoma: Analysis of Resectability and Outcomes in 380 Patients. J. Am. Coll. Surg..

[45] Bird N., Elmasry M., Jones R., Elniel M., Kelly M., Palmer D., Fenwick S., Poston G., Malik H. (2016). Role of staging laparoscopy in the stratification of patients with perihilar cholangiocarcinoma. BJS.

[46] Neuhaus P., Thelen A., Jonas S., Puhl G., Denecke T., Veltzke-Schlieker W., Seehofer D. (2011). Oncological Superiority of Hilar En Bloc Resection for the Treatment of Hilar Cholangiocarcinoma. Ann. Surg. Oncol..

[47] Nimura Y., Hayakawa N., Kamiya J., Kondo S., Shionoya S. (1990). Hepatic segmentectomy with caudate lobe resection for bile duct carcinoma of the hepatic hilus. World J. Surg..

[48] Hong S.S., Han D.H., Choi G.H., Choi J.S. (2019). Comparison study for surgical outcomes of right versus left side hemihepatectomy to treat hilar cholangiocellular carcinoma. Ann. Surg. Treat. Res..

[49] Nagino M., Ebata T., Yokoyama Y., Igami T., Sugawara G., Takahashi Y., Nimura Y. (2013). Evolution of Surgical Treatment for Perihilar Cholangiocarcinoma A Single-Center 34-Year Review of 574 Consecutive Resections. Ann. Surg..

[50] Meyer C.G., Penn I., James L. (2000). Liver transplantation for cholangiocarcinoma: Results in 207 patients. Transplantation.

[51] Robles R., Figueras J., Turrión V.S., Margarit C., Moya Á., Varo E., Calleja J., Valdivieso A., Valdecasas J.C.G., Lopez P. (2004). Spanish Experience in Liver Transplantation for Hilar and Peripheral Cholangiocarcinoma. Ann. Surg..

[52] Seehofer D., Thelen A., Neumann U.P., Denecke T., Kamphues C., Pratschke J., Jonas S., Neuhaus P., Veltzke-Schlieker W. (2009). Extended bile duct resection liver and transplantation in patients with hilar cholangiocarcinoma: Long-term results. Liver Transplant..

[53] Heimbach J.K., Gores G.J., Haddock M.G., Alberts S.R., Nyberg S.L., Ishitani M.B., Rosen C.B. (2004). Liver Transplantation for Unresectable Perihilar Cholangiocarcinoma. Semin. Liver Dis..

[54] Foo M.L., Gunderson L.L., Bender C.E., Buskirk S.J. (1997). External radiation therapy and transcatheter iridium in the treatment of extrahepatic bile duct carcinoma. Int. J. Radiat. Oncol..

[55] Rosen C.B., Heimbach J.K., Gores G.J. (2010). Liver transplantation for cholangiocarcinoma. Transpl. Int..

[56] Murad S.D., Kim W.R., Harnois D.M., Douglas D.D., Burton J., Kulik L.M., Botha J.F., Mezrich J.D., Chapman W.C., Schwartz J.J. (2012). Efficacy of neoadjuvant chemoradiation, followed by liver transplantation, for perihilar cholangiocarcinoma at 12 US centers. Gastroenterology.

[57] Kloek J.J., Van Delden O.M., Erdogan D., Kate F.J.T., Rauws E.A., Busch O.R., Gouma D.J., Van Gulik T. (2008). Differentiation of malignant and benign proximal bile duct strictures: The diagnostic dilemma. World J. Gastroenterol..

[58] Rosen C.B., Murad S.D., Heimbach J.K., Nyberg S.L., Nagorney D.M., Gores G.J. (2012). Neoadjuvant Therapy and Liver Transplantation for Hilar Cholangiocarcinoma: Is Pretreatment Pathological Confirmation of Diagnosis Necessary?. J. Am. Coll. Surg..

[59] Gores G.J., Gish R., Sudan D.L., Rosen C.B. (2006). MELD Exception Study Group Model for end-stage liver disease (MELD) exception for cholangiocarcinoma or biliary dysplasia. Liver Transplant..

[60] Sudan D.L., Deroover A., Chinnakotla S., Fox I., Shaw B., McCashland T., Sorrell M., Tempero M., Langnas A. (2002). Radiochemotherapy and transplantation allow long-term survival for nonresectable hilar cholangiocarcinoma. Arab. Archaeol. Epigr..

[61] Mantel H.T.J., Westerkamp A.C., Adam R., Bennet W.F., Seehofer D., Settmacher U., Sanchez-Bueno F., Prous J.F., Boleslawski E., Friman S. (2016). Strict Selection Alone of Patients Undergoing Liver Transplantation for Hilar Cholangiocarcinoma Is Associated with Improved Survival. PLoS ONE.

[62] Mansour J.C., Aloia T.A., Crane C.H., Heimbach J.K., Nagino M., Vauthey J.-N. (2015). Hilar Cholangiocarcinoma: Expert consensus statement. HPB.

[63] De Jong M.C., Nathan H., Sotiropoulos G.C., Paul A., Alexandrescu S., Marques H.P., Pulitanò C., Barroso E., Clary B.M., Aldrighetti L. (2011). Intrahepatic Cholangiocarcinoma: An International Multi-Institutional Analysis of Prognostic Factors and Lymph Node Assessment. J. Clin. Oncol..

[64] Tan J.C.C., Coburn N.G., Baxter N.N., Kiss A., Law C. (2007). Surgical Management of Intrahepatic Cholangiocarcinoma—A Population-Based Study. Ann. Surg. Oncol..

[65] Sotiropoulos G.C., Bockhorn M., Sgourakis G., Brokalaki E.I., Molmenti E.P., Neuhäuser M., Radtke A., Wohlschlaeger J., Baba H.A., Broelsch C.E. (2008). R0 Liver Resections for Primary Malignant Liver Tumors in the Noncirrhotic Liver: A Diagnosis-Related Analysis. Dig. Dis. Sci..

[66] Endo I., Gonen M., Yopp A.C., Dalal K.M., Zhou Q., Klimstra D., Dangelica M., DeMatteo R.P., Fong Y., Schwartz L. (2008). Intrahepatic cholangiocardnoma—Rising frequency, improved survival, and determinants of outcome after resection. Ann. Surg..

[67] Petrowsky H., Hong J. (2009). Current Surgical Management of Hilar and Intrahepatic Cholangiocarcinoma: The Role of Resection and Orthotopic Liver Transplantation. Transplant. Proc..

[68] Yoh T., Cauchy F., Le Roy B., Seo S., Taura K., Hobeika C., Dokmak S., Farges O., Gelli M., Cunha A.S. (2019). Prognostic value of lymphadenectomy for long-term outcomes in node-negative intrahepatic cholangiocarcinoma: A multicenter study. Surgery.

[69] Lee G., Gamblin T.C., Fong Z.V., Ferrone C.R., Goyal L., Lillemoe K.D., Blaszkowsky L.S., Tanabe K.K., Qadan M. (2019). Facility Type is Associated with Margin Status and Overall Survival of Patients with Resected Intrahepatic Cholangiocarcinoma. Ann. Surg. Oncol..

[70] Si A., Li J., Yang Z., Xia Y., Yang T., Lei Z., Cheng Z., Pawlik T.M., Lau W.Y., Shen F. (2019). Impact of Anatomical Versus Non-anatomical Liver Resection on Short-and Long-Term Outcomes for Patients with Intrahepatic Cholangiocarcinoma. Ann. Surg. Oncol..

[71] Lee A.J., Chun Y.S. (2018). Intrahepatic cholangiocarcinoma: The AJCC/UICC 8th edition updates. Chin. Clin. Oncol..

[72] Goere D., Wagholikar G.D., Pessaux P., Carrere N., Sibert A., Vilgrain V., Sauvanet A., Belghiti J. (2006). Utility of staging laparoscopy in subsets of biliary cancers—Laparoscopy is a powerful diagnostic tool in patients with intrahepatic and gallbladder carcinoma. Surg. Endosc..

[73] Reames B.N., Ejaz A., Koerkamp B.G., Alexandrescu S., Marques H.P., Aldrighetti L., Maithel S.K., Pulitanò C., Bauer T.W., Shen F. (2017). Impact of major vascular resection on outcomes and survival in patients with intrahepatic cholangiocarcinoma: A multi-institutional analysis. J. Surg. Oncol..

[74] Le Roy B., Gelli M., Pittau G., Allard M.-A., Pereira B., Serji B., Vibert E., Castaing D., Adam R., Cherqui D. (2017). Neoadjuvant chemotherapy for initially unresectable intrahepatic cholangiocarcinoma. BJS.

[75] Wu L., Tsilimigras D.I., Paredes A.Z., Mehta R., Hyer J.M., Merath K., Sahara K., Bagante F., Beal E.W., Shen F. (2019). Trends in the Incidence, Treatment and Outcomes of Patients with Intrahepatic Cholangiocarcinoma in the USA: Facility Type is Associated with Margin Status, Use of Lymphadenectomy and Overall Survival. World J. Surg..

[76] Ratti F., Cipriani F., Ariotti R., Gagliano A., Paganelli M., Catena M., Aldrighetti L. (2015). Safety and feasibility of laparoscopic liver resection with associated lymphadenectomy for intrahepatic cholangiocarcinoma: A propensity score-based case-matched analysis from a single institution. Surg. Endosc..

[77] Wei F., Wang G., Ding J., Dou C., Yu T., Zhang C. (2019). Is It Time to Consider Laparoscopic Hepatectomy for Intrahepatic Cholangiocarcinoma? A Meta-Analysis. J. Gastrointest. Surg..

[78] Martin S.P., Drake J., Wach M.M., Ruff S., Diggs L.P., Wan J.Y., Brown Z.J., Ayabe R.I., Glazer E.S., Dickson P.V. (2019). Laparoscopic Approach to Intrahepatic Cholangiocarcinoma is Associated with an Exacerbation of Inadequate Nodal Staging. Ann. Surg. Oncol..

[79] Kinoshita H., Sakai K., Hirohashi K., Igawa S., Yamasaki O., Kubo S. (1986). Preoperative portal vein embolization for hepatocellular carcinoma. World J. Surg..

[80] Clavien P., Petrowsky H., DeOliveira M.L., Graf R. (2007). Strategies for safer liver surgery and partial liver transplantation. N. Engl. J. Med..

[81] Li J., Moustafa M., Linecker M., Lurje G., Capobianco I., Baumgart J., Ratti F., Rauchfuss F., Balci D., Fernandes E. (2020). ALPPS for Locally Advanced Intrahepatic Cholangiocarcinoma: Did Aggressive Surgery Lead to the Oncological Benefit? An International Multi-Center Study. Ann. Surg. Oncol..

[82] Bednarsch J., Czigany Z., Lurje I., Strnad P., Bruners P., Ulmer T.F., Dulk M.D., Lurje G., Neumann U.P. (2019). The role of ALPPS in intrahepatic cholangiocarcinoma. Langenbecks Arch. Surg..

[83] Yamashita S., Sakamoto Y., Yamamoto S., Takemura N., Omichi K., Shinkawa H., Mori K., Kaneko J., Akamatsu N., Arita J. (2017). Efficacy of Preoperative Portal Vein Embolization Among Patients with Hepatocellular Carcinoma, Biliary Tract Cancer, and Colorectal Liver Metastases: A Comparative Study Based on Single-Center Experience of 319 Cases. Ann. Surg. Oncol..

[84] Ebata T., Hirano S., Konishi M., Uesaka K., Tsuchiya Y., Ohtsuka M., Kaneoka Y., Yamamoto M., Ambo Y., Shimizu Y. (2018). Randomized clinical trial of adjuvant gemcitabine chemotherapyversusobservation in resected bile duct cancer. BJS.

[85] Ribero D., Pinna A.D., Guglielmi A., Ponti A., Nuzzo G., Giulini S.M., Aldrighetti L., Calise F., Gerunda G.E., Tomatis M. (2012). Italian Intrahepatic, C., Surgical Approach for Long-term Survival of Patients With Intrahepatic Cholangiocarcinoma A Multi-institutional Analysis of 434 Patients. Arch. Surg.

[86] Goldstein R.M., Stone M., Tillery G.W., Senzer N., Levy M., Husberg B.S., Gonwa T., Klintmalm G. (1993). Is liver transplantation indicated for cholangiocarcinoma?. Am. J. Surg..

[87] Becker N.S., Rodriguez J.A., Barshes N.R., O’Mahony C.A., Goss J.A., Aloia T.A. (2007). Outcomes Analysis for 280 Patients with Cholangiocarcinoma Treated with Liver Transplantation Over an 18-year Period. J. Gastrointest. Surg..

[88] Sapisochin G., de Lope C.R., Gastaca M., de Urbina J., Suarez M.A., Santoyo J., Castroagudin J.F., Varo E., Lopez-Andujar R., Palacios F. (2014). “Very Early” Intrahepatic Cholangiocarcinoma in Cirrhotic Patients: Should Liver Transplantation Be Reconsidered in These Patients?. Am. J. Transplant..

[89] Hong J.C., Jones C.M., Duffy J.P., Petrowsky H., Farmer D.G., French S., Finn R., Durazo F., Saab S., Tong M.J. (2011). Comparative Analysis of Resection and Liver Transplantation for Intrahepatic and Hilar Cholangiocarcinoma. Arch. Surg..

[90] Sapisochin G., Facciuto M., Rubbia-Brandt L., Marti J., Mehta N., Yao F., Vibert E., Cherqui D., Grant D., Hernandez-Alejandro R. (2016). Liver transplantation for “very early” intrahepatic cholangiocarcinoma: International retrospective study supporting a prospective assessment. Hepatology.

[91] E Lunsford K., Javle M., Heyne K., Shroff R.T., Abdel-Wahab R., Gupta N., Mobley C.M., Saharia A., Victor D.W., Nguyen D.T. (2018). Liver transplantation for locally advanced intrahepatic cholangiocarcinoma treated with neoadjuvant therapy: A prospective case-series. Lancet Gastroenterol. Hepatol..

[92] Wong M., Kim J., George B., Eriksen C., Pearson T., Robbins J., Zimmerman M.A., Hong J.C. (2019). Downstaging Locally Advanced Cholangiocarcinoma Pre-Liver Transplantation: A Prospective Pilot Study. J. Surg. Res..

[93] Braconi C., Roessler S., Kruk B., Lammert F., Krawczyk M., Andersen J.B. (2019). Molecular perturbations in cholangiocarcinoma: Is it time for precision medicine?. Liver Int..

[94] Rizvi S., Gores G.J. (2017). Emerging molecular therapeutic targets for cholangiocarcinoma. J. Hepatol..

[95] Sia D., Hoshida Y., Villanueva A., Roayaie S., Ferrer-Fabrega J., Tabak B., Peix J., Sole M., Tovar V., Alsinet C. (2013). Integrative molecular analysis of intrahepatic cholangiocarcinoma reveals 2 classes that have different outcomes. Gastroenterology.

[96] Andersen J.B., Spee B., Blechacz B.R., Avital I., Komuta M., Barbour A.P., Conner E.A., Gillen M.C., Roskams T., Roberts L.R. (2011). Genomic and genetic characterization of cholangiocarcinoma identifies therapeutic targets for tyrosine kinase inhibitors. Gastroenterology.

[97] Edeline J., Bonnetain F., Phelip J.-M., Watelet J., Hammel P., Joly J.-P., Benabdelghani M., Fartoux L., Bouhier-Leporrier K., Jouve J.-L. (2017). LBA29Adjuvant GEMOX for biliary tract cancer: Updated relapse-free survival and first overall survival results of the randomized PRODIGE 12-ACCORD 18 (UNICANCER GI) phase III trial. Ann. Oncol..

[98] Primrose J.N., Fox R., Palmer D.H., Prasad R., Mirza D., Anthoney D.A., Corrie P., Falk S., Wasan H.S., Ross P.J. (2017). Adjuvant capecitabine for biliary tract cancer: The BILCAP randomized study. J. Clin. Oncol..

[99] Shroff R.T., Kennedy E.B., Bachini M., Bekaii-Saab T., Crane C., Edeline J., El-Khoueiry A., Feng M., Katz M.H., Primrose J.N. (2019). Adjuvant Therapy for Resected Biliary Tract Cancer: ASCO Clinical Practice Guideline. J. Clin. Oncol..

[100] Valle J.W., Wasan H., Palmer D.H., Cunningham D., Anthoney A., Maraveyas A., Madhusudan S., Iveson T., Hughes S., Pereira S.P. (2010). Cisplatin plus Gemcitabine versus Gemcitabine for Biliary Tract Cancer. N. Engl. J. Med..

[101] Okusaka T., Nakachi K., Fukutomi A., Mizuno N., Ohkawa S., Funakoshi A., Nagino M., Kondo S., Nagaoka S., Funai J. (2010). Gemcitabine alone or in combination with cisplatin in patients with biliary tract cancer: A comparative multicentre study in Japan. Br. J. Cancer.

[102] Valle J.W., Furuse J., Jitlal M., Beare S., Mizuno N., Wasan H., Bridgewater J., Okusaka T. (2014). Cisplatin and gemcitabine for advanced biliary tract cancer: A meta-analysis of two randomised trials. Ann. Oncol..

[103] Sama A.R., Denlinger C.S., Vogel A., He A.R., Bousmans N., Zhang W., Walgren R.A., Valle J.W. (2017). Gemcitabine and cisplatin plus ramucirumab or merestinib or placebo in first-line treatment for advanced or metastatic biliary tract cancer: A double-blind, randomized phase II trial. J. Clin. Oncol..

[104] Valle J.W., Wasan H., Lopes A., Backen A.C., Palmer D.H., Morris K., Duggan M., Cunningham D., Anthoney D.A., Corrie P. (2015). Cediranib or placebo in combination with cisplatin and gemcitabine chemotherapy for patients with advanced biliary tract cancer (ABC-03): A randomised phase 2 trial. Lancet Oncol..

[105] Malka D., Cervera P., Foulon S., Trarbach T., De La Fouchardière C., Boucher E., Fartoux L., Faivre S., Blanc J.-F., Viret F. (2014). Gemcitabine and oxaliplatin with or without cetuximab in advanced biliary-tract cancer (BINGO): A randomised, open-label, non-comparative phase 2 trial. Lancet Oncol..

[106] Lamarca A., Hubner R.A., Ryder W.D., Valle J.W. (2014). Second-line chemotherapy in advanced biliary cancer: A systematic review. Ann. Oncol..

[107] Walter T., Horgan A.M., McNamara M.G., McKeever L., Min T., Hedley D., Serra S., Krzyzanowska M.K., Chen E., Mackay H. (2013). Feasibility and benefits of second-line chemotherapy in advanced biliary tract cancer: A large retrospective study. Eur. J. Cancer.

[108] Bridgewater J., Palmer D., Cunningham D., Iveson T., Gillmore R., Waters J., Harrison M., Wasan H., Corrie P., Valle J.W. (2012). Outcome of second-line chemotherapy for biliary tract cancer. Eur. J. Cancer.

[109] Lamarca A., Palmer D.H., Wasan H.S., Ross P.J., Ma Y.T., Arora A., Falk S., Gillmore R., Wadsley J., Patel K. (2019). ABC-06 vertical bar A randomised phase III, multi-centre, open-label study of active symptom control (ASC) alone or ASC with oxaliplatin / 5-FU chemotherapy (ASC plus mFOLFOX) for patients (pts) with locally advanced / metastatic biliary tract cancers (ABC) previously-treated with cisplatin/gemcitabine (CisGem) chemotherapy. J. Clin. Oncol..

[110] Lamarca A., Ross P., Wasan H.S., A Hubner R., McNamara M.G., Lopes A., Manoharan P., Palmer D., Bridgewater J., Valle J.W. (2019). Advanced intrahepatic cholangiocarcinoma: Post-hoc analysis of the ABC-01, -02 and -03 clinical trials. J. Natl. Cancer Inst..

[111] Borad M.J., Champion M.D., Egan J.B., Liang W.S., Fonseca R., Bryce A.H., McCullough A.E., Barrett M.T., Hunt K., Patel M.D. (2014). Integrated Genomic Characterization Reveals Novel, Therapeutically Relevant Drug Targets in FGFR and EGFR Pathways in Sporadic Intrahepatic Cholangiocarcinoma. PLoS Genet..

[112] Katoh M. (2018). Fibroblast growth factor receptors as treatment targets in clinical oncology. Nat. Rev. Clin. Oncol..

[113] Javle M., Lowery M.A., Shroff R.T., Weiss K.H., Springfeld C., Borad M.J., Ramanathan R.K., Goyal L., Sadeghi S., Macarulla T. (2018). Phase II Study of BGJ398 in Patients With FGFR-Altered Advanced Cholangiocarcinoma. J. Clin. Oncol..

[114] Mazzaferro V., El-Rayes B.F., Busset M.D.D., Cotsoglou C., Harris W.P., Damjanov N., Masi G., Rimassa L., Personeni N., Braiteh F. (2018). Derazantinib (ARQ 087) in advanced or inoperable FGFR2 gene fusion-positive intrahepatic cholangiocarcinoma. Br. J. Cancer.

[115] Abou-Alfa G., Sahai V., Hollebecque A., Vaccaro G., Melisi D., Al-Rajabi R., Paulson A.S., Borad M.J., Gallinson D., Murphy A.G. (2019). Pemigatinib for Previously Treated Locally Advanced or Metastatic Cholangiocarcinoma. Lancet Oncol..

[116] Goyal L., Saha S.K., Liu L.Y., Siravegna G., Leshchiner I., Ahronian L.G., Lennerz J.K., Vu P., Deshpande V., Kambadakone A. (2016). Polyclonal secondary FGFR2 mutations drive acquired resistance to FGFR inhibition in patients with FGFR2 fusion-positive cholangiocarcinoma. Cancer Discov..

[117] A Lowery M., A Burris H., Janku F., Shroff R.T., Cleary J.M., Azad N.S., Goyal L., A Maher E., Gore L., Hollebecque A. (2019). Safety and activity of ivosidenib in patients with IDH1-mutant advanced cholangiocarcinoma: A phase 1 study. Lancet Gastroenterol. Hepatol..

[118] Bang Y., Doi T., De Braud F., Piha-Paul S., Hollebecque A., Razak A.A., Lin C., Ott P., He A., Yuan S. (2015). 525 Safety and efficacy of pembrolizumab (MK-3475) in patients (pts) with advanced biliary tract cancer: Interim results of KEYNOTE-028. Eur. J. Cancer.

[119] Bang Y.-J., Ueno M., Malka D., Chung H.C., Nagrial A., Kelley R.K., Piha-Paul S.A., Ros W., Italiano A., Nakagawa K. (2019). Pembrolizumab (pembro) for advanced biliary adenocarcinoma: Results from the KEYNOTE-028 (KN028) and KEYNOTE-158 (KN158) basket studies. J. Clin. Oncol..

[120] Le D.T., Durham J.N., Smith K.N., Wang H., Bartlett B.R., Aulakh L.K., Lu S., Kemberling H., Wilt C., Luber B.S. (2017). Mismatch repair deficiency predicts response of solid tumors to PD-1 blockade. Science.

[121] Ikeda M., Ueno M., Morizane C., Kobayashi S., Ohno I., Kondo S., Okano N., Kimura K., Asada S., Namba Y. (2019). A multicenter, open-label, phase I study of nivolumab alone or in combination with gemcitabine plus cisplatin in patients with unresectable or recurrent biliary tract cancer. J. Clin. Oncol..

[122] Arkenau H.-T., Martin-Liberal J., Calvo E., Penel N., Krebs M.G., Herbst R.S., Walgren R.A., Widau R.C., Mi G., Jin J. (2018). Ramucirumab Plus Pembrolizumab in Patients with Previously Treated Advanced or Metastatic Biliary Tract Cancer: Nonrandomized, Open-Label, Phase I Trial (JVDF). Oncology.

[123] Ioka T., Ueno M., Oh D.-Y., Fujiwara Y., Chen J.-S., Doki Y., Mizuno N., Park K., Asagi A., Hayama M. (2019). Evaluation of safety and tolerability of durvalumab (D) with or without tremelimumab (T) in patients (pts) with biliary tract cancer (BTC). J. Clin. Oncol..

[124] Macias R.I., Kornek M., Rodrigues P.M., Paiva N., Castro R.E., Urban S., Pereira S.P., Cadamuro M., Rupp C., Loosen S.H. (2019). Diagnostic and prognostic biomarkers in cholangiocarcinoma. Liver Int..

[125] Fridman W.H., Pagès F., Sautès-Fridman C., Galon J. (2012). The immune contexture in human tumours: Impact on clinical outcome. Nat. Rev. Cancer.

[126] Macias R.I., Bañales J., Sangro B., Muntané J., A Avila M., Lozano E., Perugorria M.J., Padillo F.J., Bujanda L., Marin J.J. (2018). The search for novel diagnostic and prognostic biomarkers in cholangiocarcinoma. Biochim. Biophys. Acta Mol. Basis Dis..

[127] Crespo G., Trota N., Londoño M.-C., Mauro E., Baliellas C., Castells L., Castellote J., Tort J., Forns X., Navasa M. (2018). The efficacy of direct anti-HCV drugs improves early post-liver transplant survival and induces significant changes in waiting list composition. J. Hepatol..

